# Decoding coronary physiology: towards standardized interpretation through machine learning

**DOI:** 10.1093/ehjdh/ztaf045

**Published:** 2025-04-30

**Authors:** Ioannis Skalidis, Philippe Garot, Thomas Hovasse

**Affiliations:** Institut Cardiovasculaire Paris-Sud, Hôpital Jacques Cartier, Ramsay-Santé, 6 Avenue du Noyer Lambert, Massy 91300, France; Institut Cardiovasculaire Paris-Sud, Hôpital Jacques Cartier, Ramsay-Santé, 6 Avenue du Noyer Lambert, Massy 91300, France; Institut Cardiovasculaire Paris-Sud, Hôpital Jacques Cartier, Ramsay-Santé, 6 Avenue du Noyer Lambert, Massy 91300, France

The ability to classify physiological patterns of coronary artery disease (CAD) represents a pivotal step toward personalized revascularization. Despite advances in coronary physiology and its integration into clinical trials and guidelines, the field continues to rely on expert-driven, visually interpreted characterizations of focal, diffuse, and complex lesion morphologies. This paradigm introduces variability and hampers reproducibility, particularly outside specialized centres. The recent integration of machine learning (ML) models into angiography-derived physiological assessment offers a novel pathway toward improved reproducibility and standardization.

The recent work by Zhu *et al*.^1^ introduces an ML-based framework trained on angiography-derived physiological data to classify CAD patterns.^1^ This represents an important methodological inflection point. By combining μFR virtual pullback data with multivariate functional features and supervised learning, the authors move beyond operator-dependent interpretation and dichotomous thresholds, proposing a model with potential for broader generalizability. Several aspects of this study open important avenues for reflection and discussion.

First, the effort to formalize physiological pattern classification via ML highlights the ongoing challenge of defining reliable reference standards. The study uses consensus from an expert panel as ground truth, revealing substantial interobserver variation—even among experienced physiologists. This highlights both the difficulty of the task and the opportunity that data-driven tools present. At the same time, reliance on expert consensus, while necessary, underscores the absence of an external, outcome-linked benchmark for physiological classification. As ML tools increasingly interface with clinical decision-making, future studies may consider anchoring model development and validation to procedural or prognostic endpoints rather than interpretive consensus alone.^2,3^

Second, the limitations of fixed-threshold strategies such as PPGi ≥ 0.78 are made explicit. The finding that threshold-based classification underperforms ML models trained on continuous and composite features reflects a broader truth in cardiovascular medicine: biological patterns rarely conform to binary cut-offs. The use of multivariate functional principal component analysis within the model framework is particularly notable, allowing integration of spatial and functional vessel characteristics in a mathematically coherent way. This approach opens the door for more dynamic, individualized classification paradigms, tailored to the patient and the lesion, rather than a single index.^4^

Third, the discussion around model interpretability is timely and necessary. Penalized logistic regression provides a more transparent alternative to black-box algorithms, offering clinicians insight into the variables driving prediction. This is an important consideration for integration into procedural workflows, where time constraints and user confidence demand models that are not only accurate, but explainable. Random forest models, while slightly more performant in multiclass tasks, may benefit from similar efforts at explainability, such as feature importance mapping or surrogate modelling.^5^

Fourth, the generalizability of the proposed models warrants further study. Although developed on data from two European centres, real-world implementation will require validation across broader, more heterogeneous populations. Differences in angiographic quality, acquisition protocols, and disease burden may influence model behaviour. Moreover, the extent to which these classification outputs correlate with downstream outcomes—functional PCI success, symptom relief, or long-term events—remains to be established.^6,7^

Finally, the choice to frame the classification task in binary and multiclass terms (focal vs. diffuse; focal vs. non-focal; focal vs. diffuse vs. mixed/serial) is both clinically and methodologically pragmatic. However, this structure raises broader questions about how to best define actionable categories for intervention. While these labels offer a starting point, future iterations of such models might explore outcome-oriented categories—for example, physiologically optimisable vs. non-optimisable vessels—more directly linked to clinical impact.

The study by Zhu *et al*.^1^ is a timely and commendable effort toward data-informed, standardized physiological assessment. As coronary physiology continues to evolve beyond the measurement of numerical indices and into the realm of pattern-based interpretation, tools such as this are essential. They not only support clinical decision-making but also provide a substrate for more personalized and reproducible revascularization strategies.

Future work should aim to embed such models within prospective decision pathways, to test their impact on clinical outcomes, and to validate their robustness across settings. The field stands to benefit from continued discussion on the methodological underpinnings, clinical targets, and system-level integration of ML-based physiology tools.
